# Monitoring of Post-match Fatigue in Professional Soccer: Welcome to the Real World

**DOI:** 10.1007/s40279-018-0935-z

**Published:** 2018-05-08

**Authors:** Christopher Carling, Mathieu Lacome, Alan McCall, Gregory Dupont, Franck Le Gall, Ben Simpson, Martin Buchheit

**Affiliations:** 1 Institute of Coaching and Performance, University of Central Lancashire, Greenbank Building, Preston, PR1 2HE UK; 2 Performance Department, Paris Saint-Germain Football Club, Saint-Germain-en-Laye, France; 3 Research Department for Sports and Exercise Science, Faculty of Health, Life and Social Sciences, Edinburgh Napier University, Edinburgh, UK; 4 Medical Department, Arsenal Football Club, London, UK; 5 Federation Française de Football, Paris, France

## Abstract

Participation in soccer match-play leads to acute and transient subjective, biochemical, metabolic and physical disturbances in players over subsequent hours and days. Inadequate time for rest and regeneration between matches can expose players to the risk of training and competing whilst not entirely recovered. In professional soccer, contemporary competitive schedules can require teams to compete in excess of 60 matches over the course of the season with periods of fixture congestion occurring, prompting much attention from researchers and practitioners to the monitoring of fatigue and readiness to play. A comprehensive body of research has investigated post-match acute and residual fatigue responses. Yet the relevance of the research for professional soccer contexts is debatable, notably in relation to the study populations and designs employed. Monitoring can indeed be invasive, expensive, time inefficient, and difficult to perform routinely and simultaneously in a large squad of regularly competing players. Uncertainty also exists regarding the meaningfulness and interpretation of changes in fatigue response values and their functional relevance, and practical applicability in the field. The real-world need and cost–benefit of monitoring must be carefully weighed up. In relation to professional soccer contexts, this opinion paper intends to (1) debate the need for post-match fatigue monitoring; (2) critique the real-world relevance of the current research literature; (3) discuss the practical burden relating to measurement tools and protocols, and the collection, interpretation and application of data in the field; and (4) propose future research perspectives.

## Key Points

**Table:** 

Uncertainty exists around the real-world impact of research regarding post-match fatigue (PMF) monitoring and its usefulness in informing readiness to play in professional soccer players.
Practitioners must carefully weigh up the need and cost–benefit for monitoring PMF and requirements should be determined on a case-by-case basis.
Fatigue monitoring requires a more practical approach using data derived in training sessions and the development of tools to enable the simultaneous, instantaneous and non-invasive capture of multiple sources of information during and following play.

## Introduction

Participation in soccer match-play leads to acute and transient subjective, biochemical, metabolic and physical disturbances in players over subsequent hours and days [1–3]. Inadequate time for rest and regeneration between matches can expose players to the risk of training and competing whilst not entirely recovered. In professional soccer, contemporary competitive schedules can require teams to compete in excess of 60 matches over the course of the season with periods of fixture congestion occurring, prompting much attention from researchers and practitioners to the monitoring of fatigue [4]. Accordingly, teams systematically monitor post-match fatigue (PMF) using a variety of methods and tools in an attempt to evaluate recovery and determine their readiness status for ensuing training and competition [1].

In our opinion, the real-world necessity to monitor PMF systematically in professional soccer should be debated for several reasons. For example, doubts subsist regarding the extent to which players are actually exposed to periods of match congestion [5]. A review of the literature on match congestion also showed that competitive performance is generally unaffected in professional players competing in a minimum of 75 min play across successive matches played over a short time period (e.g. two matches in a single week), potentially questioning the real-world need for monitoring [6]. Similarly, to our knowledge there is no evidence that incomplete physical, physiological and/or psychological recovery status actually causes players to underperform in ensuing match-play.

Justification for tracking PMF in professional club settings founded on findings previously reported in the scientific literature should also be debated in relation to the ecological validity of the populations commonly investigated (e.g. non-elite players) and reality of the experimental scenarios used. The practical difficulties in systematically conducting monitoring (even for purely performance and non-research purposes) in professional standard performers [7] merit discussion. Coach buy-in, player compliance and logistical burden can be problematic. Finally, the limitations of tools and protocols combined with concerns relating to the real-world meaningfulness of data, their interpretation and practical application through subsequent interventions are key issues.

In relation to professional soccer contexts, this opinion paper intends to (1) debate the need for PMF monitoring; (2) critique the real-world relevance of the current research literature; (3) discuss the practical burden relating to measurement tools and protocols, and the collection, interpretation and application of data in the field; and (4) propose future research perspectives.

## Debating the Real-World Need for Monitoring Post-match Fatigue (PMF)

A key issue in soccer concerns the competitive schedules of professional soccer clubs. The majority do not participate in international club competitions and only play a single game per week. Do schedules simply, therefore, render redundant, whether partly or entirely, the need for systematic PMF monitoring, particularly in view of future match performance? In contrast, some clubs are regularly exposed to short (e.g. three games in 8 days) and/or extended periods of fixture congestion (e.g. eight games in 1 month). However, analysis of a professional club regularly participating in European club competitions showed its players were spared extensive exposure to such congested schedules despite high availability for selection [5]. The authors suggested that squad rotation strategies restricted exposure to competition when players were potentially not fully recovered following the previous match. Research in additional club settings is nevertheless required to verify this finding.

Professional soccer players in the most successful clubs can still be required to play on a bi-weekly basis over the course of the season. On occasions, therefore, incomplete physical, physiological and/or psychological recovery could occur [1]. Yet, to our knowledge, there are no data available reporting that players with incomplete recovery in one or more of these performance areas actually suffer from a decrease in running or skill-related performance in ensuing match-play. If coaching practitioners obtain match performance data demonstrating that players are coping ‘physically’ (e.g. maintaining high-speed running activity), despite a potentially greater hidden internal load, and ‘technically’ (e.g. consistent passing accuracy), then they can legitimately question the extent of player fatigue, hence the need for PMF monitoring. Investigations conducted in professional soccer generally show that match-to-match running and technical outputs are unaffected in players competing in consecutive games in a short timeframe [6], for example, when performing for over 75 min on two occasions in a single week. A key question arises: do professional players need to be fully recovered to produce the physical and technical outputs required to respond to game demands? Analysis of elite Scandinavian male soccer players over three matches played in 1 week reported highest values for running performance in the third game despite increased pre-match values (albeit non-significant) for inflammatory and muscle damage markers compared with those obtained prior to the first match [8]. Similarly, do professional players’ natural physical ‘qualities’ and/or ‘robustness’ offer protection against fatigue and enable quick recovery rates? Recent work in professional soccer has reported an association between lower body strength and power production and post-match recovery potential [9]. It is also reasonable to suggest that players adopt pacing strategies in an attempt to maintain performance and reduce the magnitude of fatigue [10].

To summarise, the necessity to track PMF seems questionable from a purely match performance-related perspective. In our experience, sports science practitioners tend to place more emphasis on the prescription of PMF tracking in an attempt to reduce the risk of non-contact injury, which is substantially greater when the time interval between matches is short [11]. It is, however, worth noting that anecdotal evidence collected by the present authors suggests that injury risk in relation to game load is highly individual, and may be more player- than load-dependent.

## Critique of Current Literature: Research Lacks Relevance for Professional Soccer Settings

An extensive body of evidence exists on the acute and residual fatigue responses in soccer players following match-play [1–3]. Yet, in our opinion, the true worth of the literature in relation to professional settings is questionable for two main reasons, which are discussed in turn in Sects. 3.1 and 3.2.

### Playing Standard

A compilation of research across various standards of play, including amateur, semi-professional and professional players [2], reported that a 72 h time interval is generally necessary to completely restore balance in the majority of subjective and objective fatigue-related markers, although some might remain affected up to 120 h post-match [1]. Caution is necessary, however, when making inferences from data derived in studies investigating populations of differing playing standards. Indeed, this time interval to achieve full recovery might not truly reflect responses in professional-standard players. A case study in an Italian professional team showed that 48 h sufficed to ensure complete recovery in several objective and subjective fatigue-related markers [12]. A sub-analysis of PMF responses collated across different playing standards in a recent review [3] using values solely derived from the professional-standard populations cited is necessary. Similarly, information regarding the best- (home match, fresh players, no match congestion) versus worst-case (travel, fatigued before starting, congestion) time to recovery span scenarios would be useful.

### One-Off Datasets

In general, studies tend to investigate fatigue responses following a single match [12–18]. Repeated measures gathered at different phases of the season [19] and following multiple consecutive matches played over a short timeframe [8, 20] are scarce. ‘One-off’ data, for example, do not account for the recognised large match-to-match variation in physical demands [21], which might lead to inaccurate benchmark profiling of fatigue responses. The possible isolated and combined effects of travel (e.g. duration, time zones), kick-off time, ‘current form’ and changes in own and opponent’s playing systems and tactics should be accounted for where possible to ensure future study designs are in sync with real-world competition scenarios. Recent research in professional [22] and elite under 23 players [23] has shown strong associations between match result, opponent standard, and game location and subjective measures of well-being. Similarly, collective data for the team as a whole are also generally reported for these one-match scenarios. Yet large player intra-variability in responses exists [24], which again can be associated with contextual differences across matches. Unfortunately, there is a general lack of information relating to context surrounding the findings reported across the current literature.

An additional issue concerns the overlap of acute exercise-induced and chronic changes (due to accumulation of exercise loads from the weeks or months before) in fatigue responses to a given match [25]. Caution is again necessary when interpreting findings from single-match studies. The effects of training (e.g. season phase, maintenance phase, individual programmes, layoff/rehabilitation period) and prior match exposure (games/minutes played, fixture congestion) render interpretation of one-off changes in values reported across the literature difficult. Consensus is generally lacking on an appropriate number of measures, season phase and timespan for collection to build a valid benchmark profile for making confident comparisons of changes in any given fatigue marker and their true association with match performance. Despite these difficulties in discerning the nature of the fatigue, isolated data still provide a picture of players’ current status, and whenever monitoring is possible, practitioners can continually build up player profiles over time to provide a range of values for comparison.

## Conducting PMF Monitoring in the Professional Soccer Club Setting

### Is There Actually a Time and Place for PMF Monitoring?

In our opinion and experience there is a frequent disconnect between opportunities to monitor and what can actually be achieved in practice. The experimental scenarios used in the scientific literature are unrepresentative of and unrealistic for application in professional settings. For instance, research commonly examines ‘acute’ fatigue responses in the 24 h period following match-play [3]. Yet in the event of a typical one-match week, participating players frequently have a rest day following competition. Therefore, fatigue monitoring in the acute phase is not always feasible.

During two-match weeks, PMF data can in theory again inform workload adjustment and evaluate readiness for ensuing competition. However, the realities of between-match preparation frequently reduce any potential impact. The 24- to 72-h period post-match coincides with the preparation phase leading into the next match. The day after matches, clubs tend to conduct post-match recovery modalities (e.g. cold water therapy) in an attempt to alleviate fatigue and quicken recovery [26]. These recovery processes are prioritised over the collection of information on fatigue [7]. While players are usually on-site, gathering data in the interval between successive matches can be logistically difficult. Travel and match preparation—the latter including team talks, video sessions, short tactical training sessions, in-day sleep strategies and media duties for certain players—considerably reduce opportunities for monitoring. Also, on the second day following competition, coaching practitioners generally want every player on the training pitch to prepare collectively for the forthcoming match, disregarding any individual requirements. The timing of kick-offs in certain matches can affect opportunities to monitor markers at the same timepoints typically used in the literature (e.g. match + 24 and + 72 h) and restrict comparisons with existing findings. If measures have been obtained in the acute post-competition phase, data could, in theory, be used to make inferences about the magnitude of fatigue over the following 48- to 72-h period if further data collection is not possible for the mentioned reasons. However, attempting to predict fatigue or responses in certain variables at + 72 h based on + 24 h values is challenging. Recovery status, in our experience, is influenced by a myriad of factors including previous match locomotor activity, the use or not of post-competition recovery strategies (e.g. ice baths, nutrition), individual physical characteristics and/or training workload between match + 24 and + 72 h.

Another practical burden is that assessments of PMF are considered necessary for every participating player due to the considerable inter-individual differences in fatigue-related responses and recovery potential [1]. Given the logistical burden as well as availability and willingness of players (and that of coach and other support staff) to participate, this is difficult. Yet sports science practitioners typically accept what data they can obtain, irrespective of the number of players the information has been collected on, to gain an idea of the team response and, at best, attempt to tailor recovery in these players.

Finally, a combination of metrics is recommended to enable holistic interpretation of acute and residual fatigue status, which is multifactorial in nature [4]. Anticipation of fatigue prior to forthcoming match-play can be challenging if a limited number of measures are available. Residual responses in certain markers vary in relevance at later timepoints [4], due in part to previous match locomotor activity [27]. For example, jump performance can require 48 h to recover fully, while perceptions of fatigue might persist at 72 h [28]. In our experience, practitioners are only able to collect two or three measures due to the aforementioned practical burden. However, they can still tailor their recovery modalities to at least target the fatigued system(s) they have data on. Conversely, if several measures are available, the time and resources necessary to collate, clean, analyse, interpret and report the data can be considerable despite advances in software that automate processes [29].

### Critical Appraisal of Tools and Protocols for Collecting Data

There can be considerable burden due to the number of staff and resources required to run daily operations and difficulties are frequently encountered regarding the tools and protocols that are available to practitioners and commonly used in scientific research. The biochemical and metabolic procedures employed in research are considered expensive even by key stakeholders within clubs at the very highest standards of the game. Players are reluctant to accept blood or saliva sampling as this is considered invasive. Sampling also requires specialist equipment and training, although portable and user-friendly devices now exist. In addition, biological markers are prone to a considerable intra-assay and inter-assay variability and consensus on the optimal or practically most relevant biological parameter has not yet been reached [30]. Time of collection, diet and presence of injury influence biochemical responses [31]. Other tools including nerve stimulation, electromyography and muscle function analyses have been used to explore fatigue [28]. However, due to user and athlete burden, it is unlikely these tools can be routinely and simultaneously employed in a large squad of players. Moreover, laboratory-based assessments clearly cannot be employed in the field so it is difficult to verify in professional players the information that is frequently provided by current research.

A recent review [2] identified a large range of field-based physical testing methods for examining PMF, including repeated sprint and intermittent endurance assessments. Unfortunately, these tests frequently place intense physical demands on already ‘fatigued’ players and performing multiple assessments over the recovery period is evidently impossible. The validity of repeated sprint ability tests in representing the real-world demands of the game is also debatable [32]. Nevertheless, we concede that findings have added to the literature base, especially as similar investigations cannot be performed at professional standards of play. As an alternative, submaximal versions of exhaustive tests implemented as part of a standardised warm-up can provide relevant information on training status [33]. Similarly, assessments such as a countermovement jump (CMJ) on a portable platform are quick and easy means of determining neuromuscular fatigue. Yet, in applied settings, there can be reluctance by coaches, support staff and players themselves to perform such tests as they are explosive in nature and require maximal effort and additional loading. These factors reduce applicability even when testing is performed conveniently following a customary warm-up prior to training. Motivating individuals not to perform assessments as a token gesture is also essential but not easy in practice, while practitioners must ensure players do not alter mechanics in an attempt to maximise jump performance [31]. Finally, consensus is necessary on the choice of variables measured during jump testing. For example, the ratio of flight time to contraction time is shown to be a more sensitive measure of recovery compared to jump height in professional soccer players performing a CMJ [34].

As soccer is a sport mainly involving horizontal motions, sprint testing might be a more appropriate means for evaluating real-world performance rather than jump assessments. However, short straight-line sprint performance (e.g. 10–20 m) has recently been shown to lack sensitivity as a post-match (24–48 h) indicator of physical fatigue in semi-professional soccer players [28]. Analysis of decrements in maximal velocity capability in soccer players over longer sprint distances (> 30 m) is suggested to improve evaluation of fatigue following match-play [35]. Again, constraints related to sprint testing (e.g. injury risk, additional fatigue, player compliance, coach ‘buy-in’, place of test within the day-to-day working practices) need to be carefully weighed up.

The association between post-exercise fatigue and skill-related performance has been examined using controlled assessments such as the Loughborough soccer passing and shooting tests [3]. The former specifically lacks feasibility [36] and no information is available on its validity for assessing in-game passing performance, which might be affected by the non-controlled effects of crowd and match context as well as mental fatigue potentially caused by fast ever-changing game dynamics. In our experience, professional players are simply unwilling to perform skill-related tests and practitioners would never even contemplate their usage.

An alternative to these tools and protocols is to collect external workload data derived from time–motion analyses in the preceding match and make inferences about PMF. However, the technology frequently employed has methodological limitations, especially for key variables associated with neuromuscular fatigue (e.g. acceleration, decelerations and high-speed running) [37]. For instance, commonly used optical-based player tracking systems do not provide information on force load and stride characteristics to assess the neuromuscular and mechanical demands of play. Therefore, they do not allow direct associations to be made with PMF responses from jump tests. While global positioning systems (GPS) enable collection of such data and are permitted in competition, players can be reluctant to wear devices. Also, there is contrasting evidence on correlation strength between match running indicators and muscle damage and neuromuscular performance observed at 48 h after a match [13, 27]. The pertinence of time–motion metrics in anticipating PMF might be limited to the first 24 h after match-play (when players are often resting or in recovery) and caution is necessary if these are used to inform training load or readiness status for competition thereafter [38]. Determining critical match load thresholds using these technologies to inform subsequent recovery status is also difficult due to the large inter-individual variability in workload distribution. Players complete relatively more or less low-speed activity, high-speed running, accelerations, decelerations and changes of direction than peers yet produce the same absolute match load, mainly due to differences in playing position, tactics and physical characteristics [34].

Self-reports permit collection of subjective perceptions of fatigue and well-being during the post-match phase. These are easily administered and scientifically legitimate alternatives to objective measures [39]. Yet in our experience some players are reluctant to provide information on their perceptions post-match. Opportunities for data collection are frequently result-dependent and findings may not reflect true perceptions following a loss or a poor performance. Player education and language barriers, and changes in collection methods, timing or the practitioner conducting the monitoring can confound the problem [7]. Self-reporting is influenced by outside influences (e.g. expectations of supporters and media) [40] and individuals might answer in a ‘socially desirable’ manner during intensive competitive schedules, over-reporting favourable responses and under-reporting unfavourable responses to appear to be coping [41].

### Functional Relevance and Real-World Meaningfulness of Data and Their Application

A key concern is the functional relevance and real-world meaningfulness of changes in PMF responses during the recovery period. Accounting for technical and biological test measurement error so that meaningful decrements (e.g. ‘red flags’) in fatigue and performance can be distinguished from natural variations in measurements is evidently a key issue [24]. Some practitioners might use pre-set cut-off thresholds (e.g. defined as the smallest worthwhile change [SWC], arbitrary ± 5–10% or, more correctly, 0.2 of between-players standard deviation [SD] or fraction/multiples of individual SD, depending on the variables of interest [42]) for detecting meaningful changes. Yet we can ask, for example, what would be the real-world effect of a 2.8% reduction (i.e. greater than the SWC) in CMJ peak power output (PPO) values reported at 48 h post-match reported in reserve team professional soccer players [43] on the proportion of duels won/lost in a match played shortly after?

In general, the degree to which PMF data, even if collected robustly in a standardised and reliable way, are actually employed in practice to modify subsequent training delivery is unknown. Anecdotal evidence reports that information can inform adjustments of training workload to ensure players are not under- or over-loaded in the lead-up to ensuing matches. A simple subjective measure of muscle soreness conducted 36–48 h following match-play can aid decision-making on readiness status for a typical mid-week high-intensity aerobic conditioning session or conversely indicate the need for an additional recovery day [44]. However, how do practitioners weigh up the cost versus benefit between allowing a player an additional half- or full-days rest or missing a key tactical training session, for example, to recover a substantial 6.6% decrease in PPO derived from a CMJ 24 h following match-play (reported in the aforementioned professional reserve team players [43])? In our experience, sports science practitioners simply have no choice other than to judge changes in PMF responses on face value and make key decisions using their experience and know-how while accounting for the present context. An upskilling of staff and coaches in sport science and data analysis is arguably necessary! For additional information on identifying meaningful changes in data and decision-making consequences using monitoring systems, the reader is referred to two recent papers [24, 45].

## Research Perspectives and Monitoring Alternatives

Investigations to determine the extent to which and how PMF monitoring (question-driven and strategically implemented?) is used in professional-standard settings to impact upon daily training and selection for forthcoming competition are merited.

As regards physical testing, research using mechanical workload metrics that have a logical link with neuromuscular demands is necessary. Despite the aforementioned practical difficulties in applied settings, additional exploration of the influence of cognitive and central nervous system function, sleep behaviour, travel, season phase, nutritional status and coach feedback on PMF responses would be helpful to increase the literature base. The development of mentally fatiguing tasks with high ecological validity for soccer is essential to determine the extent to which mental fatigue occurs in players and subsequently track its time course to recovery post-match.

Future work should be directed towards using convenience data derived in training. Pilot work has shown that simple running indicators [37] and heart rate measures [46] derived from typical small-sided games can determine readiness status. Research quantifying the effects on fatigue patterns from preceding training loading and the acute:chronic workload ratio could also be worthwhile [13], although its implementation in the elite setting [47] and ability to truly predict non-contact injuries might be limited [48].

Finally, there is a need to develop tools to simultaneously, instantaneously and non-invasively capture and interpret multiple sources of information prior to, during and following training and competition. Emerging technologies such as facial tracking to evaluate well-being and smart clothing with embedded sensors providing real-time performance outputs combined with machine-learning data analysis systems hold promise once scientific legitimacy is proven.

## Conclusion

As part of the contemporary preparation process for professional soccer, fatigue monitoring post-match is conducted to evaluate player recovery and readiness to play status. Yet the real-world bedrock for systematic monitoring is debatable and need and cost–benefit must be carefully weighed up. Indeed, no evidence exists to show that match performance is actually affected in players not fully recovered, with possibly a greater case for use in injury prevention schemes. Collecting data is problematic due to staff and player buy-in and compliance as well as the logistical burden and limitations of monitoring tools and protocols. Where data are available, uncertainty exists around their real-world impact in informing ensuing workload and eventual selection for competition. While a large body of research proliferates, the populations and protocols used limit its ability to provide guidelines for application at professional standards. Finally, there is a need for more practical means of capturing and analysing multiple sources of information over the entire training and match cycle.
